# The meta-learning method for the ensemble model based on situational meta-task

**DOI:** 10.3389/fnbot.2024.1391247

**Published:** 2024-04-26

**Authors:** Zhengchao Zhang, Lianke Zhou, Yuyang Wu, Nianbin Wang

**Affiliations:** ^1^College of Computer Science and Technology, Harbin Engineering University, Harbin, Heilongjiang, China; ^2^Modeling and Emulation in E-Government National Engineering Laboratory, Harbin Engineering University, Harbin, Heilongjiang, China; ^3^School of Computer Science and Technology, Guangdong University of Technology, Guangzhou, Guangdong, China

**Keywords:** meta-learning, few-shot learning, situational meta-task, ensemble model, image recognition

## Abstract

**Introduction:**

The meta-learning methods have been widely used to solve the problem of few-shot learning. Generally, meta-learners are trained on a variety of tasks and then generalized to novel tasks.

**Methods:**

However, existing meta-learning methods do not consider the relationship between meta-tasks and novel tasks during the meta-training period, so that initial models of the meta-learner provide less useful meta-knowledge for the novel tasks. This leads to a weak generalization ability on novel tasks. Meanwhile, different initial models contain different meta-knowledge, which leads to certain differences in the learning effect of novel tasks during the meta-testing period. Therefore, this article puts forward a meta-optimization method based on situational meta-task construction and cooperation of multiple initial models. First, during the meta-training period, a method of constructing situational meta-task is proposed, and the selected candidate task sets provide more effective meta-knowledge for novel tasks. Then, during the meta-testing period, an ensemble model method based on meta-optimization is proposed to minimize the loss of inter-model cooperation in prediction, so that multiple models cooperation can realize the learning of novel tasks.

**Results:**

The above-mentioned methods are applied to popular few-shot character datasets and image recognition datasets. Furthermore, the experiment results indicate that the proposed method achieves good effects in few-shot classification tasks.

**Discussion:**

In future work, we will extend our methods to provide more generalized and useful meta-knowledge to the model during the meta-training period when the novel few-shot tasks are completely invisible.

## 1 Introduction

Deep learning (LeCun et al., 2015) has achieved great success and has become a practical method in many applications, such as computer vision (Yu et al., 2022; Jiang et al., 2023), speech recognition (Afouras et al., 2018; Zhang et al., 2019), and natural language processing (Shen et al., 2018). However, it heavily relies on a large amount of labeled training data. When the available training data is drastically reduced, traditional deep learning methods are ineffective in training. In contrast, humans can quickly learn novel tasks (i.e., few-shot tasks) through a small amount of supervised information, because people can fully apply their past learning experience to novel tasks and then can quickly adapt and learn them. We hope that artificial intelligence models can quickly learn from novel tasks with few-shot data similar to humans. This fast learning is a challenge because the artificial intelligence models must combine their previous experience with a small amount of new information while avoiding over-fitting novel tasks (Finn et al., 2017). The process of human learning has sparked our interest in the research of few-shot learning (Wang et al., 2020; Lu et al., 2023; Song et al., 2023; Zeng and Xiao, 2024) and how to fully utilize past learning experiences to few-shot tasks.

Meta-learning (Vanschoren, 2018; Elsken et al., 2020; Li et al., 2021; Liu et al., 2022; He et al., 2023; Vettoruzzo et al., 2024) was put forward to solve the problem of few-shot learning. It empowers learning systems with the ability to acquire knowledge from multiple tasks, enabling faster adaptation and generalization to new tasks (Vettoruzzo et al., 2024). Specifically, it is to provide the model, especially the deep neural network, a learning ability that allows the model to learn some meta-knowledge automatically. Meta-knowledge refers to the knowledge that can be learned outside of the model training process, such as the initial parameters of the neural network, the structure and optimizer of the neural network, and the hyperparameter of the model. In few-shot learning, meta-learning specifically refers to learning meta-knowledge from a large number of prior tasks and using them to guide the model to learn faster in novel tasks.

The meta-learning method (Hospedales et al., 2021), based on optimization, is an important branch of few-shot learning. These algorithms attempt to obtain a better initial model or correct gradient descent direction through meta-learning. It optimizes initial parameters by the meta-learner so that the learner can converge faster in novel tasks and achieve fast adaptation and learning with few-shot data.

**Figure 1:**
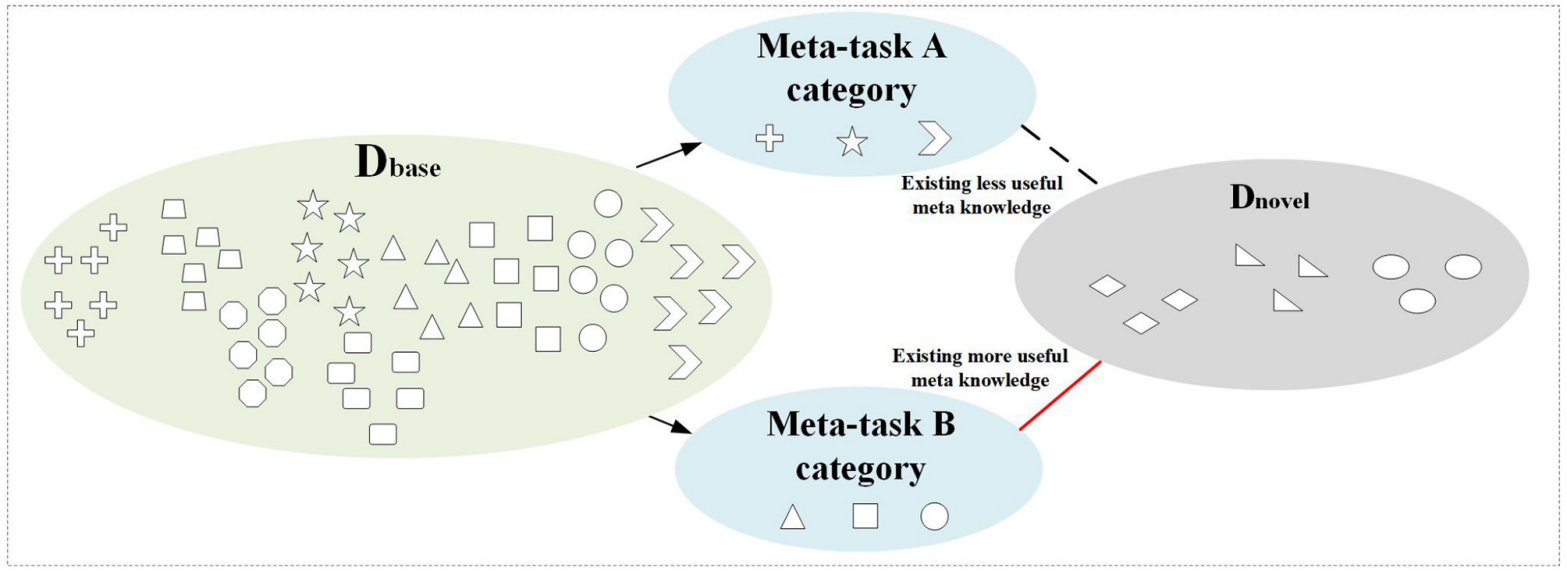
The relationship between meta-tasks selected from base class dataset and few-shot dataset.

In few-shot learning, the pre-learned base class data before learning few-shot novel tasks is crucial for the generalization ability of the model. Selecting a good base class can often greatly improve the learning efficiency of novel tasks (Zhou et al., 2020). Figure 1 shows the randomly selected meta-tasks A and B from the base class data during the meta-training period. From the perspective of the sample category feature, meta-task B has more meta-knowledge related to the few-shot task. Therefore, it is crucial to select effective meta-knowledge from the base class for few-shot tasks, which can improve the efficiency and effect of few-shot learning.

Based on the above motivation, this article argues that existing meta-learning methods do not take into account the relationship between base classes used for meta-learner learning and novel classes in few-shot tasks. During the meta-training period, this can lead to providing more irrelevant meta-knowledge for few-shot tasks, which will affect the efficiency and effect of few-shot learning in the meta-testing. Therefore, it is necessary to consider the feature relationships between base class data and few-shot data in the meta-training.

To improve this problem, first, we should select the most relevant set of candidate meta-tasks for novel tasks from the base class as much as possible to construct situational meta-tasks, which in turn provide optimal initial models for novel tasks. Then, the diversity of features among different meta-tasks can be used to promote better learning of novel tasks by multiple models.

In this article, we attempt to improve the problem of not considering relationships between base class data and few-shot novel class data by means of situational meta-task construction and multiple ensemble models. Starting from the feature relationship between base class data and few-shot data, we provide a new research idea for meta-learning. First of all, a universal feature extractor is trained in the basic learning phase to extract features of base class data and few-shot class data. Then, during the meta-training period, accurate meta-knowledge is provided for novel tasks by constructing situational meta-tasks. Furthermore, it provides good initial model parameters for novel tasks. Finally, during the meta-testing period, few-shot tasks are learned through the cooperation of multiple models. A large number of experiments on popular few-shot datasets demonstrate the effectiveness of the proposed method. Our main contributions are summarized as follows:

- This article puts forward a construction method of situational meta-task. It uses the class centroid of base class data to select the candidate meta-task sets with the stronger correlation for few-shot tasks and then sets up situational meta-tasks similar to few-shot tasks. The situational meta-tasks are the same as the few-shot tasks in form and similar to them in terms of feature. This method provides accurate and available meta-knowledge for novel tasks, which is conducive to rapid adaptation and learning on few-shot tasks.
- An ensemble model method based on meta-optimization is proposed in this article. The meta-model of situational meta-task training during meta-training is used to cooperate to complete the learning of few-shot tasks. The cooperation of multiple models improves the predictive performance and stability of a single model in the full-phase meta-learning process.
- Moreover, we extensively validate the proposed method by applying it to popular few-shot character dataset and image recognition datasets and then implementing and training through CNNs, Vgg16, and ResNet50 networks. The results indicate that the construction method of situational meta-task provides effective and available meta-knowledge for few-shot novel tasks, and the method of ensemble multiple models outperform previous state-of-the-art baselines.

## 2 Related work

The core idea of the meta-learning method is to use the past prior knowledge to guide the model to learn novel tasks. Meta-learning, as a standard approach to solving the problem of few-shot learning, which attempts to learn (Li et al., 2017). The goal of meta-learning is to enable models, especially deep neural networks, to learn how to undertake novel tasks from few-shot data. Among them, the meta-learning method based on optimization is an important branch of few-shot learning.

### 2.1 Meta-learning based on optimization

The idea of this kind of algorithm is to attempt to obtain a better initial model or correct gradient descent direction through meta-learning. Then, the initial parameters are optimized by the meta-learner. This enables the learner to converge faster in novel tasks and learn rapidly in the case of few-shot learning.

Finn et al. (2017) proposed a model agnostic meta-learning (MAML) method. First, the network is trained with the ability to extract universal features, and then further trained to adapt to novel tasks rapidly on this basis. This approach is considered model agnostic since it can be applied directly to any learning model trained by a gradient descent process.

Based on the idea of MAML, Li et al. (2017) put forward a meta-stochastic gradient descent method called meta-SGD based on LSTM. By meta-learning the initialization parameters, learning rate and updating direction, the trained model can be easily fine-tuned to adapt to novel tasks. This algorithm is significantly less difficult to train compared to LSTM. Compared with the MAML method, it improves the model capacity.

The reptile (Nichol et al., 2018) method was proposed by Nichol et.al., which updates fewer parameters at a time and saves a lot of time and memory costs. However, the algorithm cannot directly adapt to the fast learning performed by MAML.

Rajeswaran et al. (2019) proposed a meta-learning method of implicit gradient. In this method, a new loss function and a corresponding method for computing the gradient are designed, so that the gradient of the parameter can be obtained only by computing the result of the loss function without considering its specific optimization method.

The meta-learning method based on optimization is to find a better initialization model or gradient descent direction for few-shot data. However, existing methods learn directly on the base class dataset and rarely consider relationships between few-shot data and base class data. This will lead to the learning of irrelevant meta-knowledge on the base class, which is not conducive to few-shot learning. Therefore, this article first fully considers the feature relationships between base class data and few-shot data and uses it as prior knowledge to construct strongly relevant situational meta-tasks for meta-learners. Then, meta-learners use situational meta-tasks to carry out meta-learning based on optimization, thereby improving the effect of few-shot learning.

### 2.2 Ensemble learning

To overcome the problem of unreliable and unstable results from single model, ensemble learning aims to utilize the diversity among multiple models to improve the learning ability of multiple weak learners. It can produce a strong ensemble learner for better prediction performance (Ganaie et al., 2022).

Traditional ensemble learning methods include Bagging, Boosting, Stacking, decision tree-based, and random forest-based. The Bagging algorithm (Breiman, 1996) (such as bootstrap aggregation) is one of the earliest ensemble learning methods. Although it has a simple structure, it has excellent performance. The algorithm generates different training subsets by randomly changing the distribution of the training dataset, then trains individual learners with different training subsets, and finally integrates them as a whole.

The Boosting algorithm (Freund and Schapire, 1996) is an iteration method that transforms weak learners into a strong learner. It generates a strong learner that behaves almost perfectly by increasing the number of iterations. Stacking, also known as Stacked Generalization (Wolpert, 1992), refers to training a model that is used to integrate all individual learners. The model is trained with the output of these individual learners as input to obtain a final output.

Recently, the deep neural network has been integrated into the ensemble strategies. Deep neural decision forest (Kontschieder et al., 2015) is a learning method that combines convolutional neural networks (CNNs) and decision forest techniques. It introduces stochastic backpropagation of decision trees, which is then combined into a decision forest, resulting in a final model with better generalization performance. gcForest (Zhou and Feng, 2017) is a new method that combines the ensemble method with the deep neural network. Unlike the above method, it replaces the neurons with random forest models, using the output vector of each random forest as the input to the next layer.

## 3 Method

In this section, first, we explain the relevant definitions and concepts proposed for meta-task construction and meta-model ensemble (Section 3.1). Then, the motivation and idea of our proposed method are generally introduced (Section 3.2). Finally, in order to improve the problem of feature correlation between few-shot and base class, the **c**onstruction method of **s**ituational **m**eta-**t**ask (CSMT) proposed in meta-training (Section 3.3) is introduced and the **f**ull-phase **m**eta-learning **p**rocess of **m**ultiple **i**nitial **m**odel **c**ooperation (FMPMIMC) is described, which includes the basic learning phase and the meta-optimization phase (Section 3.4).

### 3.1 Problem definition and description

#### 3.1.1 Basic class dataset and novel class dataset(few-shot dataset)

The base class dataset is $D_{base}=\left\{\left(x_{base}^{i},y_{base}^{i}\right),i=1,2,...,N_{b}\right\}$, and the novel class dataset is $D_{novel}=\left\{\left(x_{novel}^{i},y_{novel}^{i}\right),i=1,2,...,N_{n}\right\}$, where *D*_*base*_∩*D*_*novel*_ = ∅. Few-shot tasks *T*_*novel*_ are randomly sampled from *D*_*novel*_. Each few-shot task includes support set and query set. The support set is $S=\left\{\left(x_{support}^{i},y_{support}^{i}\right),i=1,2,...,n\times k\right\}$, where *S*⊂*T*_*novel*_. The query set is $Q=\left\{\left(x_{query}^{i},y_{query}^{i}\right),i=1,2,...,n\times q\right\}$, where *Q*⊂*T*_*novel*_. Especially, *S*∩*Q* = ∅ and *S*⋃*Q* = *T*_*novel*_.

#### 3.1.2 Situational meta-task

Situational meta-task is a collection of tasks that have the same form (N-way K-shot) and related features to few-shot tasks. It is constructed from the data in the base class and is used in the meta-training. From the perspective of feature, it has a strong correlation with few-shot tasks. In form, it is the same as Nway-Kshot for few-shot tasks. Suppose that the base class dataset is represented as *D*_*base*_ = {*D*_1_, *D*_2_, *D*_3_, ..., *D*_*n*_} by category, and a 5way-1shot support set denotes *S* = {*x*_1_, *x*_2_, *x*_3_, *x*_4_, and *x*_5_}. By using the situational meta-task construction method, the most relevant candidate meta-task set (such as the candidate meta-task set for few-shot *x*_1_ is $D_{x_{1}}^{meta\text{\_}task}=\left\{D_{c},D_{k},D_{p},D_{q},\text{ and }D_{m}\right\}$, where *D*_*c*_, *D*_*k*_, *D*_*p*_, *D*_*q*_, and *D*_*m*_∈*D*_*base*_) is selected from the base class dataset for each category of few-shot dataset (taking few-shot *x*_1_ as an example), and then the situational meta-tasks are constructed by extracting a sample from each category of few-shot own related candidate meta-task set (such as $D_{x_{1}}^{meta\text{\_}task},D_{x_{2}}^{meta\text{\_}task},D_{x_{3}}^{meta\text{\_}task},$ $D_{x_{4}}^{meta\text{\_}task},\text{ and }D_{x_{5}}^{meta\text{\_}task}$, where $D_{x_{i}}^{meta\text{\_}task}\underset{i\neq j}{⋂}D_{x_{j}}^{meta\text{\_}task}=\emptyset$).

#### 3.1.3 Multiple initial models

During the meta-training period, the set of different meta-models is trained using different situational meta-tasks, which can be described as *M* = {*M*_1_, *M*_2_, *M*_3_, ..., *M*_*n*_}. They are used as the basis for cooperative learning in the meta-testing.

#### 3.1.4 Full-phase meta-learning process

It includes the basic learning phase and the meta-optimization phase. The basic learning phase provides a universal feature extractor for the meta-optimization phase, which is used for constructing situational meta-tasks. The meta-optimization phase includes meta-training and meta-testing. During the meta-training period, the multiple initial models are trained using situational meta-tasks. They are used to adapt and learn few-shot tasks cooperatively in the meta-testing period.

### 3.2 Overview

**Figure 2:**
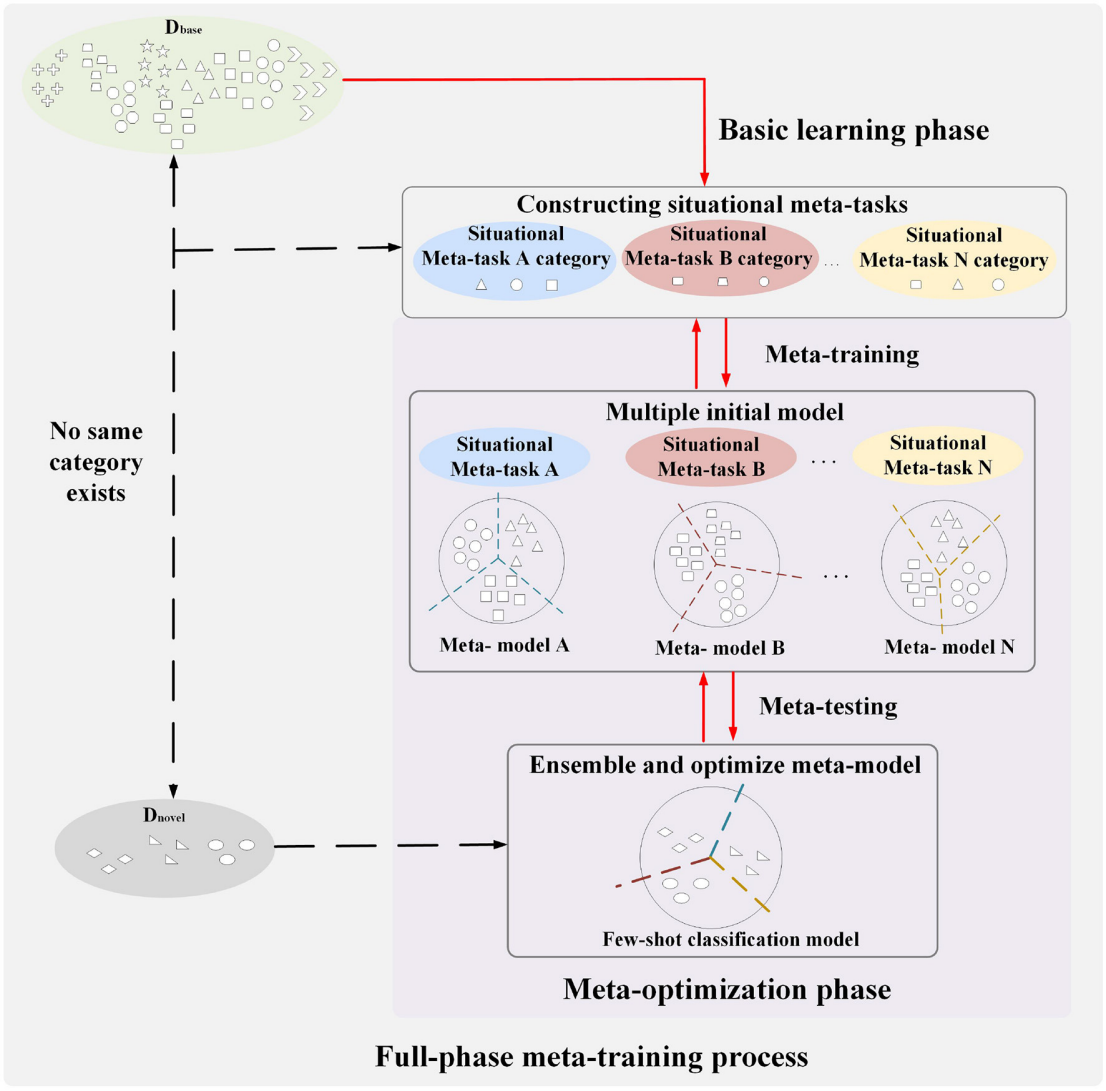
Schematic diagram of a full-phase meta-learning method based on construction of situational meta-task and cooperation with multiple initial models.

The full-phase meta-learning method based on situational meta-task construction and multiple initial model cooperation is shown in Figure 2. It consists of the basic learning phase and the meta-optimization phase. The base class data and few-shot data do not have the same category, which means that few-shot data are novel tasks for models. The general idea of our proposed method is introduced below from the process of the full-phase meta-learning.

First, in the basic learning phase, a universal feature extractor is trained for constructing situational meta-tasks in the meta-optimization phase. The purpose of constructing situational meta-tasks is to provide more effective meta-knowledge for few-shot tasks, enabling the model to rapidly adapt to few-shot tasks. Then, situational meta-tasks are used to train multiple initial models in the meta-training of the meta-optimization phase. Finally, in the meta-testing of the meta-optimization phase, the few-shot dataset is used to optimize multiple initial models, promoting cooperation among models, and more fully utilizing meta-knowledge to learn few-shot classification model.

### 3.3 A construction method of situational meta-task

The meta-learning methods based on optimization find better initial models or gradient descent directions for few-shot tasks through base class dataset. This allows models to adapt and learn quickly for few-shot tasks. However, existing methods directly learn on the base class dataset, rarely considering the feature relationships between few-shot data and base class data. This will result in learning more irrelevant meta-knowledge on the base class, which is not conducive to few-shot learning. Therefore, our research motivation is to provide relevant and effective meta-knowledge for few-shot tasks from the base class. Furthermore, it provides better initial model parameters for few-shot learning, which enables fast learning and adaptation on few-shot data.

**Figure 3:**
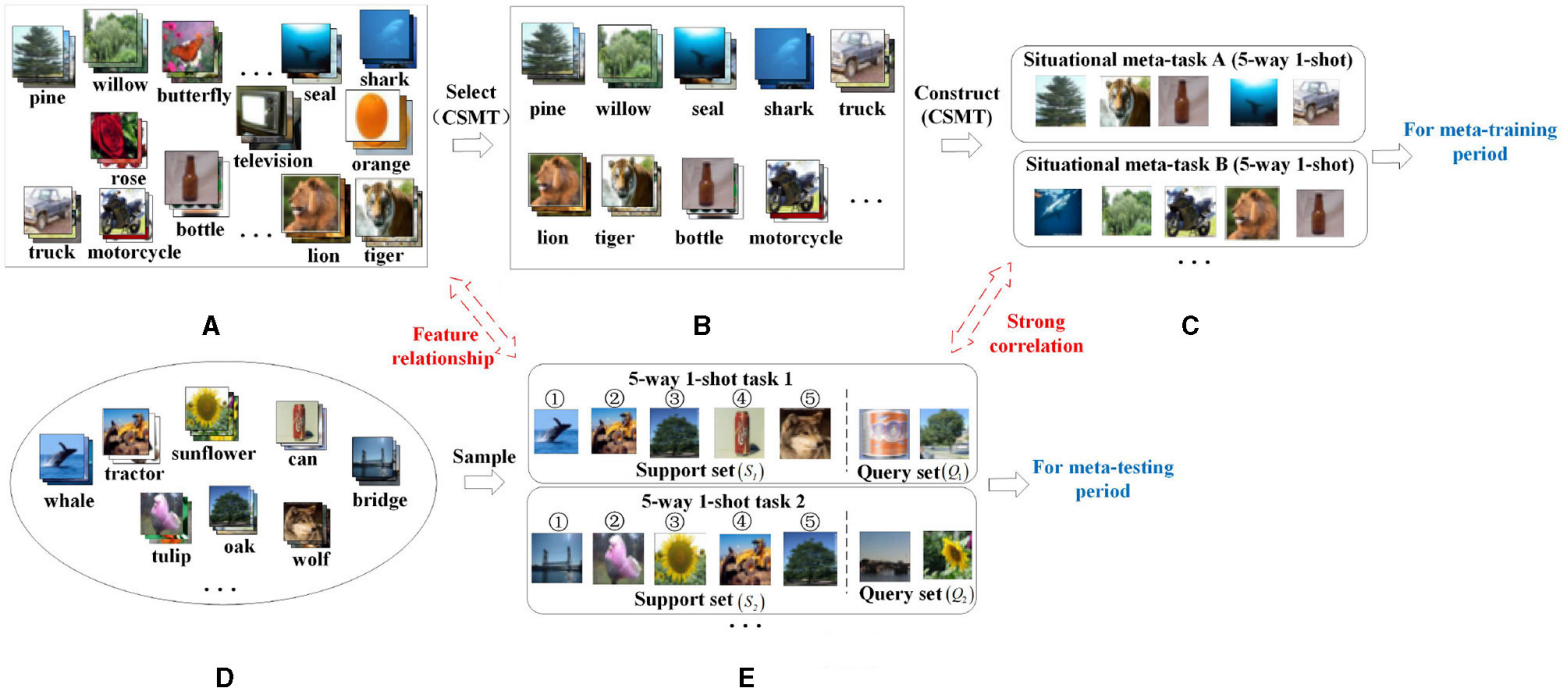
A schematic diagram of the situational meta-task construction process. **(A)** Base class dataset (*D*_*base*_). **(B)** Candidate meta-task set (*Meta*_*task*_*S*_1__). **(C)** Situational meta-task ($D_{S_{1}}^{meta\text{\_}task}$). **(D)** Few-shot database (*D*_*novel*_). **(E)** Few-shot task (*T*_*novel*_).

In order to solve the above problem, this article proposes a construction method of situational meta-task (CSMT), which is shown in Figure 3. The main idea of this method is to select the categories related to few-shot tasks from the base class dataset as candidate meta-task sets, and then use candidate meta-task sets to construct situational meta-tasks. Specifically, first, few-shot tasks *T*_*novel*_ (Figure 3E) are randomly sampled from the few-shot dataset *D*_*novel*_ (Figure 3D). Each few-shot task consists of a support set (*S*_1_, *S*_2_, ...) and a query set (*Q*_1_, *Q*_2_, ...). Then, the support set *S*_1_ (shown in the above Figure 3E) of 5-way 1-shot task 1 is used as an example to construct its situational meta-tasks. The relevant categories from the base class dataset *D*_*base*_ (Figure 3A) is selected as a candidate meta-task set using the feature relationships between the centroid $C_{base}^{i}$ of each category of the base class dataset and the centroid $C_{novel}^{j}$ of each category of the support set *S*_1_. The relevant categories from the base class dataset are selected as the candidate meta-task set *Meta*_*task*_*S*_1__ (Figure 3B). Finally, the candidate meta-task set is used to construct some situational meta-tasks $D_{S_{1}}^{meta\text{\_}task}$ (Figure 3C) for the 5-way 1-shot task 1. The situational meta-task A has the same form(5-way 1-shot) and related features to the support set *S*_1_ of the 5-way 1-shot task 1. The situational meta-tasks are used during meta-training and the few-shot tasks are used during meta-testing.

**The construction process of situational meta-task is given as follows:**

**Step 1: Computing the central support point (centroid) of each class**

The mean vector is computed for all feature vectors of each class in the base class dataset as the central support point $C_{base}^{i}$ for that class. It can be represented as Equation (1):


(1) $$\begin{array}{l} C_{\text{base}}^{i}=\frac{1}{\left|D_{\text{base}}^{i}\right|}\underset{\left(x_{\text{base}}^{\text{s}},{\text{y}}_{\text{base}}^{\text{s}}\right)\in D_{\text{base}}^{i}}{\sum }f_{\varphi }\left(x_{\text{base}}^{\text{s}}\right), \end{array}$$

where $D_{base}^{i}$ is the sample set of the ith class in the base class dataset and $x_{base}^{s}$ is the feature vector that belongs to $D_{base}^{i}$. *f*_φ_(·) is an embedding function.

Similarly, when the form of the few-shot dataset is N-way K-shot, the mean vector of all feature vectors in each category in the few-shot data set is calculated as the central support point $C_{novel}^{j}$ of the class. When the form of the few-shot dataset is N-way 1-shot, the central support point $C_{novel}^{j}$ of each category is the sample feature. It can be represented as Equation (2):


(2) $$\begin{array}{l} C_{\text{novel}}^{j}=\frac{1}{\left|K\right|}\underset{\left(x_{\text{novel}}^{\text{s}},{\text{y}}_{\text{novel}}^{\text{s}}\right)\in D_{\text{novel}}^{i}}{\sum }f_{\varphi }\left(x_{\text{novel}}^{\text{s}}\right). \end{array}$$

**Step 2: Selecting few-shot candidate meta-task sets from the base class dataset**

The feature distance (*Dis*_*j*_*i*_) between the central support point $C_{novel}^{j}$ of each category in the few-shot dataset and the central support point $C_{base}^{i}$ of each category in the base class dataset are calculated, and the distance using cosine similarity is calculated. It can be represented as Equation (3):


(3) $$\begin{array}{l} {\mathrm{Dis}}_{j_{-}i}=\cos\left(C_{\text{novel}}^{j},C_{\text{base}}^{i}\right). \end{array}$$

The calculated data are sorted in the descending order, and the top K class is selected as the candidate meta-task set for each few-shot class and is denoted as *Meta*_*task*_*j*_. It can be represented as Equation (4):


(4) $$\begin{array}{l} {Meta\text{\_}task}_{j}\leftarrow sort\left({Dis}_{j\text{\_}i}\;,K\right). \end{array}$$

**Step 3: Handling the conflict of candidate meta-task sets**

When two or more candidate meta-task sets contain the same category in the base class (assuming that the candidate meta-task sets corresponding to classes p and q of the few-shot dataset both contain class m of the base class dataset), select the few-shot class with the minimum centroid error as the optimal construction method to ensure that different few-shot classes select different candidate meta-task sets. The centroid error is the sum of the distance between all samples of a certain class in the few-shot dataset and the centroid of that class in the base class dataset. It can be represented as Equation (5):


(5) $$\begin{array}{l} L_{c}=\overset{k}{\underset{\begin{array}{c} i=1\\ x_{\text{novel}}^{i}\in {\text{D}}_{\text{novel}}^{p} \end{array}}{\sum }}f_{\varphi }\left({\text{x}}_{\text{novel}}^{i}\right)-C_{base}^{m}. \end{array}$$

Among them, *L*_*c*_ represents the centroid error between the samples $D_{novel}^{p}$ of the class p in the few-shot dataset and the class m in the base class dataset. $x_{novel}^{i}$ represents the sample in the few-shot dataset $D_{novel}^{p}$ and $C_{base}^{m}$ is the centroid of the class m in the base class dataset.

**Step 4: Constructing situational meta-tasks**

The tasks from candidate meta-task sets for each class of few-shot data are extracted and combined into situational meta-tasks in the form of Nway-Kshot which is the same as few-shot tasks. They are the training dataset in the meta-training period.

The construction method of situational meta-task is shown in Algorithm 1.

**Algorithm 1:**
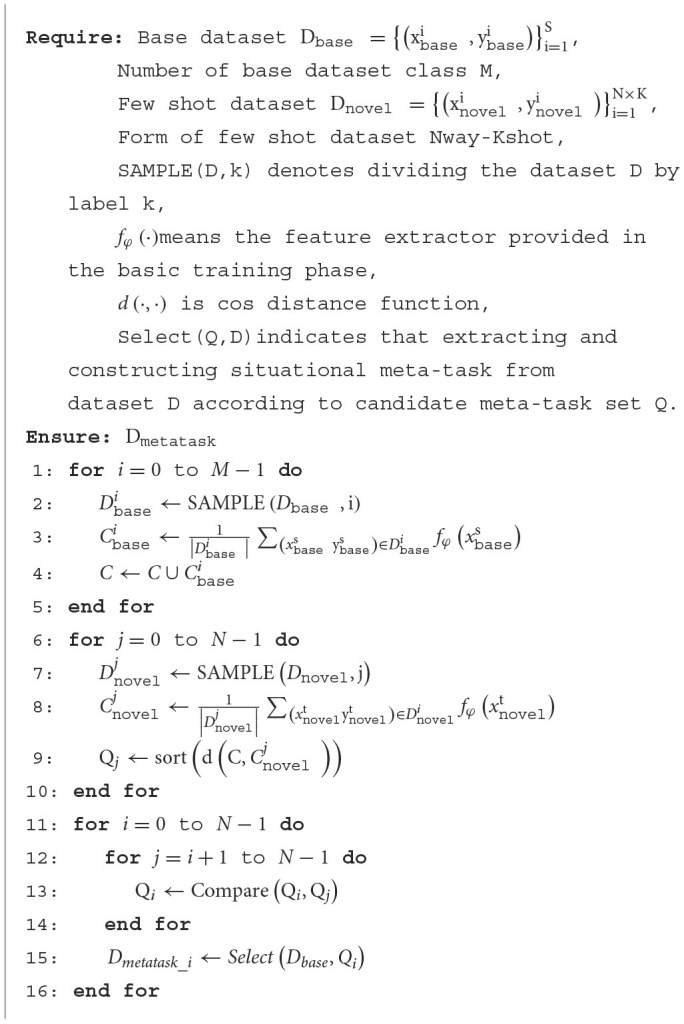
The CSMT method.

Through the situational meta-task construction method, the training dataset related to the feature of few-shot is provided for the meta-model in the meta-training. First, for each few-shot class, strongly related candidate meta-task sets are selected from the base class dataset in order to better provide useful meta-knowledge for few-shot data. Then, the candidate meta-task sets are used to construct situational meta-tasks, whose form and features are more similar to few-shot tasks, which is beneficial for the model to adapt quickly and learn novel tasks.

In this subsection, different situational meta-tasks provide models containing different meta-knowledge for few-shot tasks. Overall, this process also makes full preparation for the efficient learning of few-shot tasks in the next subsection.

### 3.4 Full-phase meta-learning process based on multiple initial model cooperation

**Figure 4:**
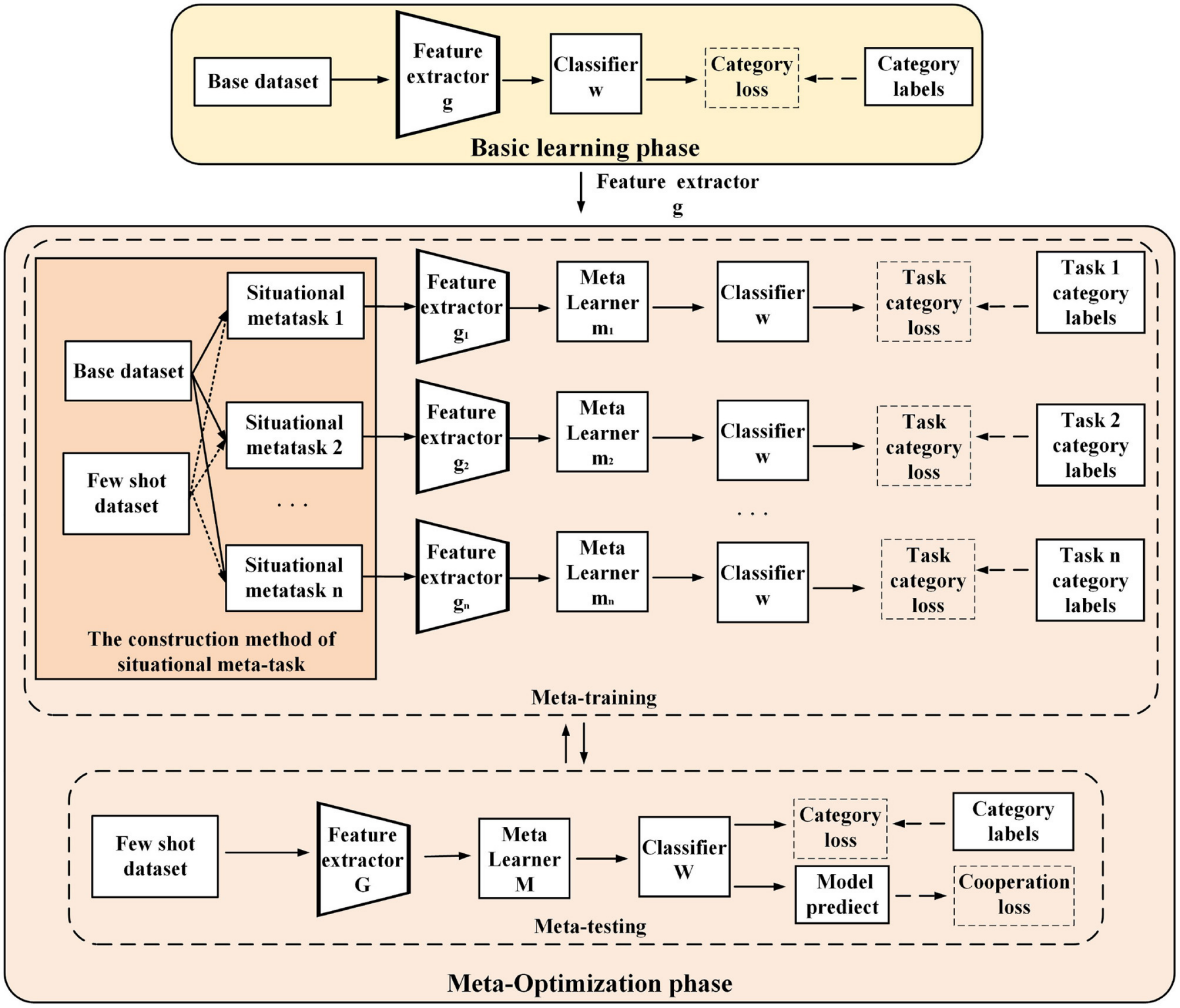
A schematic diagram of the learning process of a full-phase meta-learning method based on situational meta-task construction and cooperation with multiple initial models. In the basic training phase (above), the model learns a universal feature extractor from the base class data for situational meta-task construction. In the meta-optimization phase (below), multiple independent models are trained by situational meta-tasks in the meta-training. Then, multiple models utilize classification loss and cooperative loss to learn few-shot novel tasks in the meta-testing.

As shown in Figure 4, the full-phase meta-learning process based on multiple initial model cooperation (FMPMIMC) includes two phases: basic learning and meta-optimization. The basic learning phase provides a universal feature extractor for constructing situational meta-tasks in the meta-optimization phase.

#### 3.4.1 The basic learning phase

The model is trained by the base class data, and it can be described as Equation (6):


(6) $$L_{base\_cls}\left(f{}^{\circ}w,x_{base},y_{base}\right)=E\left[l\left(w\left(f\left(x_{base}\right)\right),y_{base}\right)\right],$$

where *L*_*base*_*cls*_ is the classification loss, *f*(·) is a feature extractor of the model, *w*(·) is a classifier, and *l*(·, ·) is a cross entropy loss function.

The basic learning phase can be analogized to the extensive human learning process, and the model gets a universal feature extractor through extensive learning. It is better to extract features in the meta-optimization phase.

#### 3.4.2 The meta-optimization phase

The meta-optimization phase includes two interactive processes: meta-training and meta-testing. First, during the meta-training period, some situational meta-tasks are constructed for few-shot tasks using the feature extractor from the base learning process (each situational meta-task contains the corresponding support set and query set).

Then, they are used to train several independent networks (each network includes components such as feature extractor, meta-learner, and classifier). The loss of each network utilizes the classified cross entropy loss of situational meta-tasks, which can be represented as Equation (7):


(7) $$\begin{array}{l} \;L_{meta\_train}\left(f{}^{\circ}m{}^{\circ}w,x_{meta\_task},y_{meta\_task}\right)\\ =E\left[l\left(w\left(m\left(f\left(x_{meta\_task}\right)\right)\right),y_{meta\_task}\right)\right], \end{array}$$

where *L*_*meta*_*train*_ is the meta-training loss of situational meta-tasks and *f*(·) is the feature extractor of the network. *m*(·) is the meta-learner and *w*(·) is the classifier. *l*(·, ·) is the cross entropy loss function of situational meta-task.

During the meta-training period, models containing diverse meta-knowledge are trained and learned on some different situational meta-tasks. During the meta-testing period, multiple initial model cooperation is used to learn novel few-shot tasks. The single model utilizes traditional cross entropy function to calculate the classification loss of the support sets in novel tasks. It can be represented as Equation (8):


(8) $$\begin{array}{l} L_{meta\_test}^{cls\_i}{\left(f_{i}{}^{\circ}m_{i}{}^{\circ}w_{i},x_{novel\_task}^{support},y_{novel\_task}^{support}\right)}_{(x_{novel\_task}^{support},y_{novel\_task}^{support})\in D_{novel\_task}^{support}}\\ \;=E\left[l\left(w_{i}\left(m_{i}\left(f_{i}\left(x_{novel\_task}^{support}\right)\right)\right),y_{novel\_task}^{support}\right)\right], \end{array}$$

where $L_{meta\text{\_}test}^{cls\text{\_}i}$ is the classification loss of a single model learning novel few-shot tasks, $x_{novel\text{\_}task}^{support}$ is the support set sample of few-shot task, and $y_{novel\text{\_}task}^{support}$ is the label of the support set sample of few-shot task.

In order to cooperate with multiple initial models to complete the learning of few-shot novel tasks, KL divergence is used between the models to calculate the difference loss in model predictions. By strengthening cooperation among models, efficient learning of few-shot novel tasks can be realized. The cooperation loss of multiple initial models can be described as Equation (9):


(9) $$\begin{array}{l} L_{meta\text{\_}test}^{dif\text{\_}i}=\frac{1}{2}\overset{n}{\underset{j=1}{\sum }}\left(KL\left({\hat{p}}_{i}||{\hat{p}}_{j}\right)+KL\left({\hat{p}}_{j}||{\hat{p}}_{i}\right)\right), \end{array}$$

where $L_{meta\text{\_}test}^{dif\text{\_}i}$ is the difference loss predicted between the ith model and other models, and ${\hat{p}}_{i}$ is the output of the softmax layer of the ith network.

During the meta-testing period, the total loss of the ith model is as follows


(10) $$\begin{array}{l} L_{meta\text{\_}test}^{i}=L_{meta\text{\_}test}^{cls\text{\_}i}+L_{meta\text{\_}test}^{dif\text{\_}i}, \end{array}$$

where $L_{meta\text{\_}test}^{i}$ is the total loss when the ith model learns few-shot tasks. $L_{meta\text{\_}test}^{cls\text{\_}i}$ is the classification loss, and $L_{meta\text{\_}test}^{dif\text{\_}i}$ is the cooperation loss between models.

The full-phase meta-learning process based on multiple initial model cooperation is shown in Algorithm 2.

**Algorithm 2:**
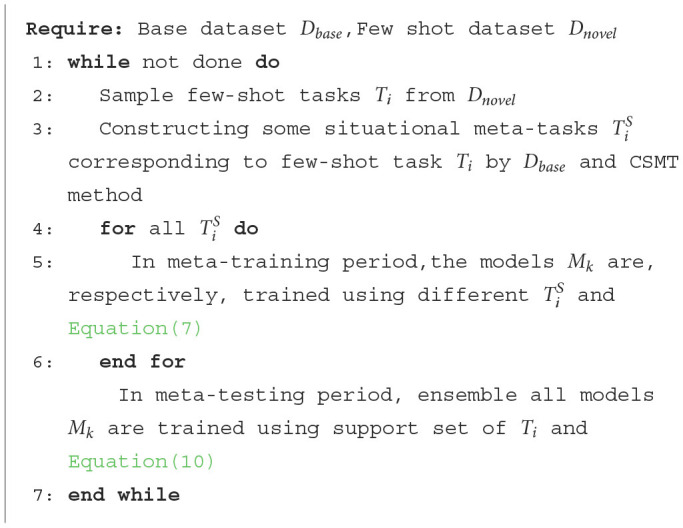
The FMPMIMC method.

In this section, a situational meta-task construction method is used to provide more relevant and effective meta-knowledge for few-shot novel tasks. During the meta-training period, different situational meta-tasks provide diverse meta-knowledge, resulting in certain differences in the meta-knowledge learned by models. During the meta-testing period, the diversity of models is used for cooperative learning few-shot novel tasks. By reducing the prediction differences among models, the prediction quality and stability of the whole model are further improved.

## 4 Experiments

In this section, first, we introduce several benchmark few-shot datasets (Omniglot, CIFAR-100 and MiniImageNet) used in our experiments (Section 4.1). Then, we conduct three experiments, namely, the situational meta-task construction experiment (Section 4.2), the classification experiment of the full-phase meta-learning multiple initial model cooperation (Section 4.3), and the related parameter setting experiment (Section 4.4). These experiments are used to evaluate CSMT and FMPMIMC methods.

### 4.1 Setup

**Figure 5:**
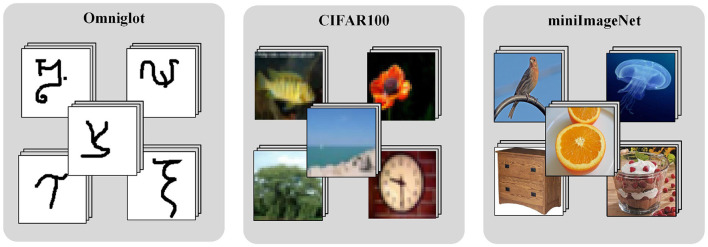
The samples of the standard dataset used in the experiments.

In this study, the performance of the proposed method is evaluated on three few-shot image classification datasets, including the simple character dataset Omniglot (Lake et al., 2011), the complex image datasets CIFAR-100 (Boris et al., 2018), and MiniImageNet (Vinyals et al., 2016). Figure 5 shows typical images for each dataset.

#### 4.1.1 Omniglot dataset

It consists of 1,623 handwritten characters (equivalent to 1,623 classes) from 50 different languages. Each class has 20 different handwritings (equivalent to 20 samples in each class). The size of each sample is 28 × 28 pixels. In each few-shot experiment, we randomly selected 100 classes as the few-shot dataset (five classes were selected multiple times as few-shot tasks), and the remaining classes were used as the base class dataset.

#### 4.1.2 CIFAR-100 dataset

It contains 100 classes, each with 600 color images of size 32 × 32 pixels. In total, 500 samples from each class are used as the training dataset, and the remaining 100 samples are used as the testing dataset. In each few-shot experiment, we randomly selected 20 classes as the few-shot dataset(5 classes were selected multiple times as few-shot tasks), and the remaining 80 classes were used as the base class dataset.

#### 4.1.3 MiniImageNet dataset

It consists of 100 classes selected from ImageNet, and each category has 600 color images with the size of 84 × 84 pixels. Among them, the training dataset, the validation dataset, and the testing dataset contain 64 classes, 16 classes and 20 classes, respectively. In each few-shot experiment, we randomly selected five classes from the testing dataset multiple times as few-shot learning tasks, and the remaining 80 classes from the training and validation datasets as the base class dataset.

### 4.2 The experiment of situational meta-task construction

**Figure 6:**
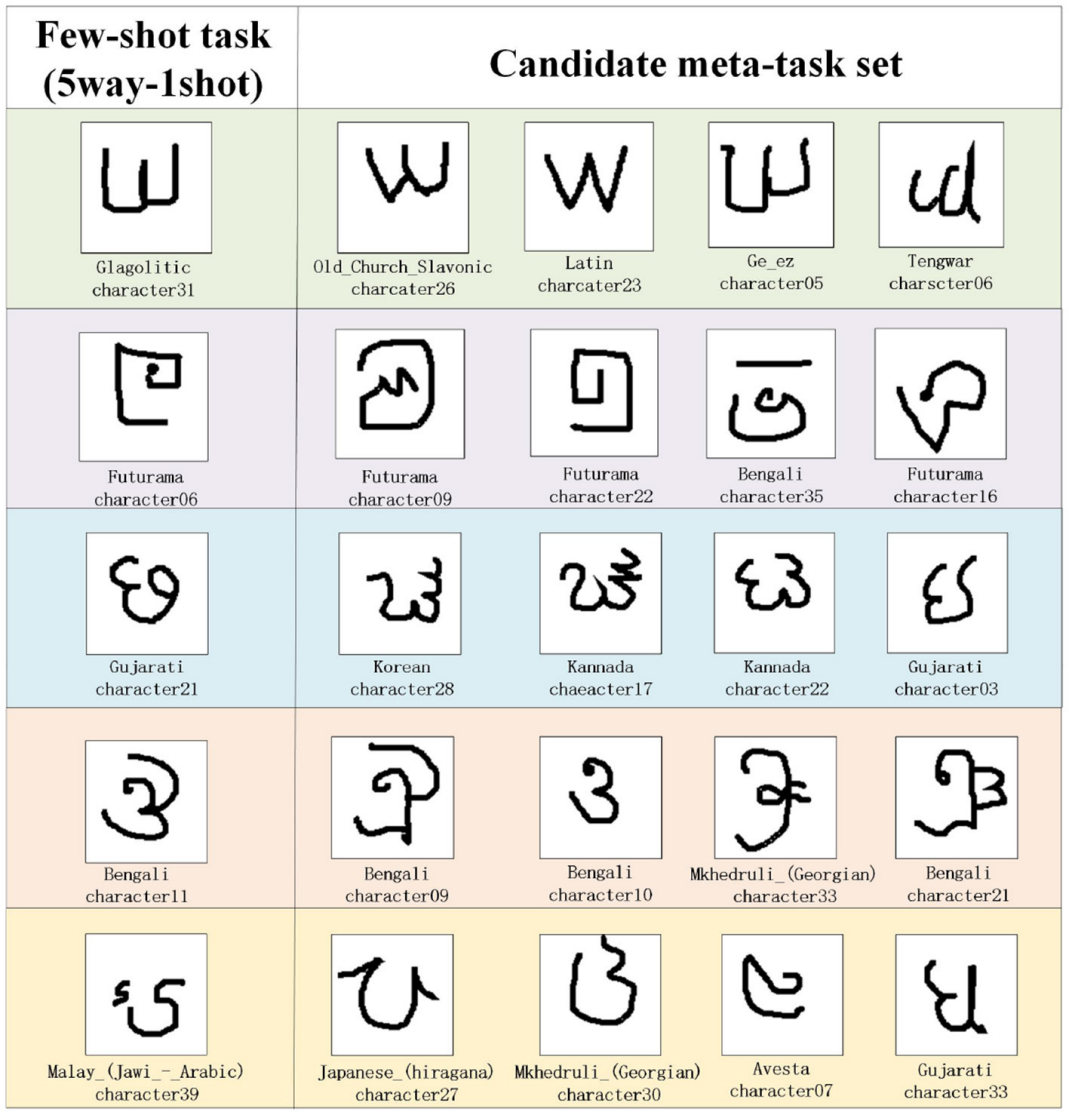
The samples of candidate meta-task set selected for the few-shot task in the Omniglot dataset.

For the construction of situational meta-task in the experiment, first, we randomly selected 1 or 5 samples from five classes as few-shot tasks (5way-1shot/5way-5shot) from the few-shot dataset for each experiment. Then, during the meta-training period, the construction method of situational meta-task is used to select candidate meta-task sets for few-shot novel tasks. Using the experiment results from the Omniglot dataset as an illustration, Figure 6 shows an example of candidate meta-task sets selected according to the 5way-1shot task. The following are the analysis of the experimental results.

**Figure 7:**
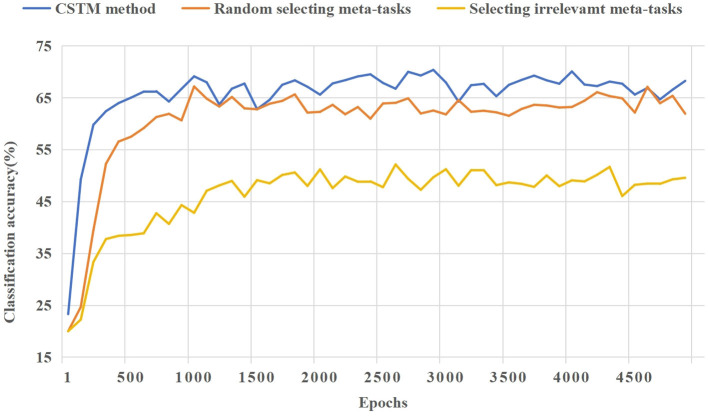
A comparison of the training process of ablation experiments for the CSMT method based on CIFAR-100.

(1) The candidate meta-task set selected for the 5way-1shot task in the Omniglot dataset is visualized in Figure 6. The features and shapes of sample classes in the candidate meta-task set are similar to those of few-shot task, and its samples can provide more useful and effective meta-knowledge for the few-shot task. Then samples are extracted corresponding to the few-shot task form (5way-1shot) to construct situational meta-tasks.
(2) The average accuracy of the 5way-1shot and 5way-5shot experiments using a single model on the Omniglot dataset by CSMT is reported in Table 1. In the 5way-1shot experiment of the Omniglot dataset, the experimental result is 0.23% higher than that of the advanced SNAIL method. This shows that the CSMT method provides more effective meta-knowledge for few-shot tasks, which is helpful for few-shot learning.
(3) Table 2 shows the average accuracy of 5way-1shot and 5way-5shot experiments of a single model on the CIFAR-100 dataset by the CSMT method. Compared with the advanced Dual TriNet method, it improves the performance by 6.16% and 1.1%. The experimental results demonstrate the effectiveness of the meta-knowledge provided for few-shot tasks during the meta-training period. The performance is outstanding in the experiment of 5-way 1-shot, which shows that the model can rapidly adapt to the learning of few-shot tasks through the training of situational meta-tasks.
(4) Figure 7 shows the ablation experiment of the CSMT method. The three cases of providing situational meta-tasks, selecting random meta-tasks, and providing irrelevant meta-tasks for the meta-model are compared. It shows the learning effect of the model on the novel task as the number of iterations increases in the meta-testing. As can be seen from Figure 7, it is important to provide effective meta-knowledge for few-shot tasks. The CSMT method enables the model to adapt to few-shot tasks more quickly.

**Table 1:** The 5-way 1-shot /5-shot CSMT classification accuracy (%) on the Omniglot dataset.

**Method**	**5-way 1-shot**	**5-way 5-shot**
MAML (Finn et al., 2017)	98.7 ± 0.4	**99.9** ± **0.1**
TCML (Mishra et al., 2017)	98.96 ± 0.2	99.75 ± 0.11
Gaussian PN (Fort, 2017)	99.07 ± 0.03	99.73 ± 0.02
Reptile (Nichol et al., 2018)	97.68 ± 0.04	99.48 ± 0.06
SNAIL (Nikhil et al., 2018)	99.07 ± 0.16	99.78 ± 0.09
R2-D2 (Bertinetto et al., 2019)	98.91 ± 0.05	99.74 ± 0.02
CSMT (ours)	**99.3** ± **0.18**	99.6 ± 0.12

The bold values are the best experimental results.

**Table 2:** The 5-way 1-shot /5-shot CSMT classification accuracy (%) on the CIFAR-100 dataset.

**Method**	**5-way 1-shot**	**5-way 5-shot**
TADAM (Boris et al., 2018)	40.10 ± 0.40	56.10 ± 0.40
MetaOptNet (Lee et al., 2019)	41.10 ± 0.60	55.50 ± 0.60
ProtoNet (Snell et al., 2017)	41.54 ± 0.76	57.08 ± 0.76
DC (Lifchitz et al., 2019)	42.04 ± 0.17	57.05 ± 0.16
Matching Nets (Vinyals et al., 2016)	43.88 ± 0.75	57.05 ± 0.71
MTL (Sun et al., 2019)	45.10 ± 1.80	57.60 ± 0.90
DeepEMD (Zhang et al., 2022)	46.47 ± 0.78	63.22 ± 0.71
DEML+Meta-SGD (Zhou et al., 2018)	61.62 ± 1.01	77.94 ± 0.74
Dual TriNet (Chen et al., 2019)	63.41 ± 0.64	78.43 ± 0.62
CSMT (ours)	**69.57** ± **1.20**	**79.53** ± **0.93**

The bold values are the best experimental results.

Through the construction method of situational meta-task, useful meta-knowledge is provided for the learning of novel tasks in the meta-testing. However, different situational meta-tasks provide different meta-knowledge for few-shot tasks. In order to make full use of the meta-knowledge of different situational meta-tasks, a full-phase meta-learning experiment with multiple initial model cooperation is carried out in the next section.

### 4.3 The experiment of the full-phase meta-learning process based on multiple initial model cooperation

In the previous subsection, the CSMT method was used to provide multiple initial models for learning few-shot novel tasks in the meta-testing. There are differences in the meta-knowledge provided by different initial models, resulting in different learning effects on novel tasks. In this subsection, the FMPMIMC method is used to reduce the differences between models and realize the rapid adaptation and efficient learning of few-shot novel tasks.

First, the CSMT method is used to provide *n* initial meta-models (*n* = 2, 3, 5, 10, and 20) for few-shot novel tasks. Then, during the meta-training period, each of the *n* initial meta-models uses its own situational meta-tasks training. During the meta-testing period, these *n* initial meta-models are trained together on corresponding few-shot tasks by the FMPMIMC method. Finally, we conduct 5way-1shot and 5way-5shot experiments on CIFAR-100 and MiniImageNet datasets, respectively. The average results of the experiments are reported as shown in Tables 3, 4. The following are the analysis of the experimental results.

**Table 3:** The 5-way 1-shot /5-shot FMPMIMC classification accuracy (%) on the CIFAR-100 dataset.

**Method**	**5-way 1-shot**	**5-way 5-shot**
TADAM (Boris et al., 2018)	40.10 ± 0.40	56.10 ± 0.40
MetaOptNet (Lee et al., 2019)	41.10 ± 0.60	55.50 ± 0.60
ProtoNet (Snell et al., 2017)	41.54 ± 0.76	57.08 ± 0.76
DC (Lifchitz et al., 2019)	42.04 ± 0.17	57.05 ± 0.16
Matching Nets (Vinyals et al., 2016)	43.88 ± 0.75	57.05 ± 0.71
MTL (Sun et al., 2019)	45.10 ± 1.80	57.60 ± 0.90
DeepEMD (Zhang et al., 2022)	46.47 ± 0.78	63.22 ± 0.71
DEML+Meta-SGD (Zhou et al., 2018)	61.62 ± 1.01	77.94 ± 0.74
Dual TriNet (Chen et al., 2019)	63.41 ± 0.64	78.43 ± 0.62
CSMT	69.57 ± 1.20	79.53 ± 0.93
FMPMIMC (without KL divergence)	70.40 ± 0.65	80.82 ± 0.70
FMPMIMC (ours)	**73.15** ± **0.53**	**83.06** ± **0.60**

The bold values are the best experimental results.

**Table 4:** The 5-way 1-shot /5-shot FMPMIMC classification accuracy (%) on MiniImagenet dataset.

**Method**	**5-way 1-shot**	**5-way 5-shot**
Matching Nets (Vinyals et al., 2016)	43.56 ± 0.84	55.31 ± 0.73
ProtoNet (Snell et al., 2017)	49.42 ± 0.78	68.20 ± 0.66
Dual TriNet (Chen et al., 2019)	58.12 ± 1.37	76.92 ± 0.69
DEML+Meta-SGD (Zhou et al., 2018)	58.49 ± 0.91	71.28 ± 0.69
TADAM (Boris et al., 2018)	58.50 ± 0.30	76.70 ± 0.30
MTL (Sun et al., 2019)	61.20 ± 1.80	75.50 ± 0.80
DC (Lifchitz et al., 2019)	62.53 ± 0.19	78.95 ± 0.13
MetaOptNet (Lee et al., 2019)	64.09 ± 0.62	80.00 ± 0.45
DeepEMD (Zhang et al., 2022)	65.91 ± 0.82	82.41 ± 0.56
SIB (Hu et al., 2020)	70.00 ± 0.40	79.20 ± 0.40
BD-CSPN (Liu et al., 2020)	70.31 ± 0.93	81.89 ± 0.60
EPNet (Rodŕıguez et al., 2020)	70.74 ± 0.85	79.20 ± 0.40
Meta-BNNet (Gao et al., 2023)	71.73 ± 0.23	82.58 ± 0.17
CSMT	71.65 ± 1.05	78.32 ± 0.93
FMPMIMC (without KL divergence)	72.05 ± 0.67	81.20 ± 0.80
FMPMIMC (ours)	**73.49** ± **0.40**	**83.55** ± **0.75**

The bold values are the best experimental results.

As shown in Table 3, the FMPMIMC method achieves the highest average classification accuracy compared with the advanced baseline methods in the 5way-1shot and 5way-5shot experiments of the CIFAR-100 dataset, with an increase of 9.74 and 4.63%. This method has improved by 1.76 and 0.97% compared to advanced Meta-BNNet methods in experiments on the MiniImageNet dataset. Meanwhile, compared with the multiple model cooperation strategy of the FMPMIMC method and the single model CSMT method, the experimental results are improved, and the model is more stable, which shows the stability and effectiveness of the FMPMIMC method. For the KL divergence term in the FMPMIMC method, we conducted ablation experiments, and the experimental results in Tables 3, 4 show the effectiveness of the KL divergence term in the loss function. The experimental results of 5way-1shot on the two datasets are more prominent, which reflects the feature of the FMPMIMC method that enables the model to rapidly adapt to few-shot tasks.

### 4.4 The parameter setting experiment

In this subsection, we first analyze the relationship between the number of ensemble models and the effect of few-shot learning and the influence of interaction frequency of meta-training and meta-testing in the meta-optimization phase. Then, the relationship between the number of tasks in the candidate meta-task set and the few-shot learning effect is explored.

#### 4.4.1 The experiment on the number of ensemble model

**Figure 8:**
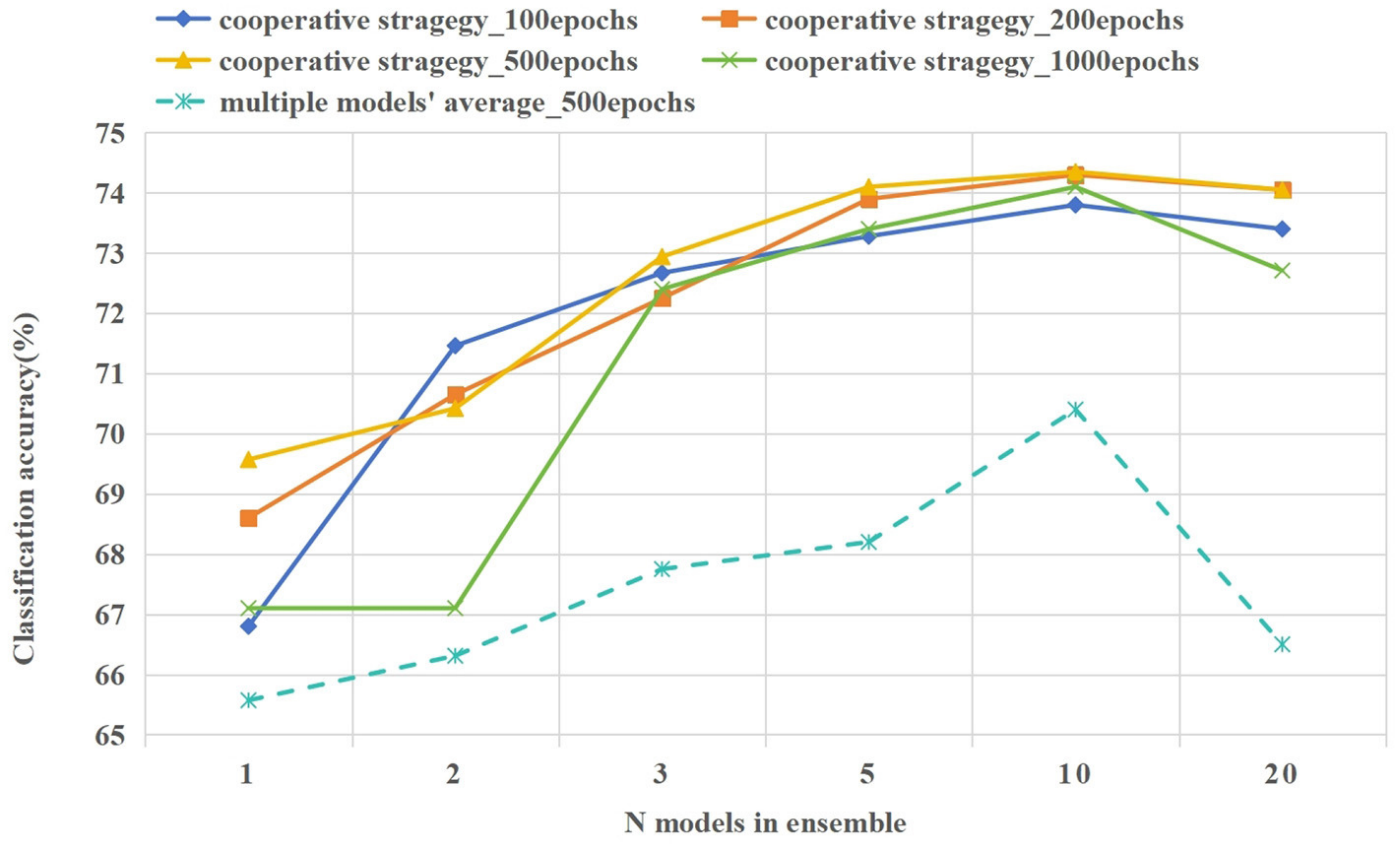
The classification accuracy of various numbers ensemble models. The solid line gives the classification accuracy after collaborative prediction of multiple models, and the average performance of a single model is plotted with a dashed line.

In the 5way-1shot experiment of the CIFAR-100 dataset, we set the number of ensemble model to 1, 2, 3, 5, 10, and 20. Meanwhile, we set the interaction frequency of meta-training and meta-testing to 100, 200, 500, and 1,000 epochs. In Figure 8, we compared the prediction accuracy using model cooperation strategy (existing KL divergence) and the average performance of multiple models(without KL divergence). The following are the analysis of the experimental results.

As shown in Figure 8, when a cooperative strategy is adopted for prediction, the accuracy of prediction improves with the increase in the number of cooperative models. When the number of models is 10, the prediction effect is the best, and the memory of multiple model ensemble is about 26 GB. When the number of models increases to 20, the performance of multiple model cooperation prediction is decreased. The performance of multiple model cooperation prediction is always better than its average performance, and its fluctuation is small, which reflects the effectiveness of the model cooperation strategy (existing KL divergence). Similarly, under the same number of ensemble models, the influence of different interaction frequencies between meta-training and meta-testing on model prediction results is explored. In many cases, the model gives the best prediction results when the interaction frequency is 500 epochs. The frequent interaction between meta-training and meta-testing will lead to overfitting of the model to few-shot data. Too little interaction frequency will lead to poor generalization of the model for few-shot tasks.

#### 4.4.2 The experiment on the number of tasks in the candidate meta-task sets

**Figure 9:**
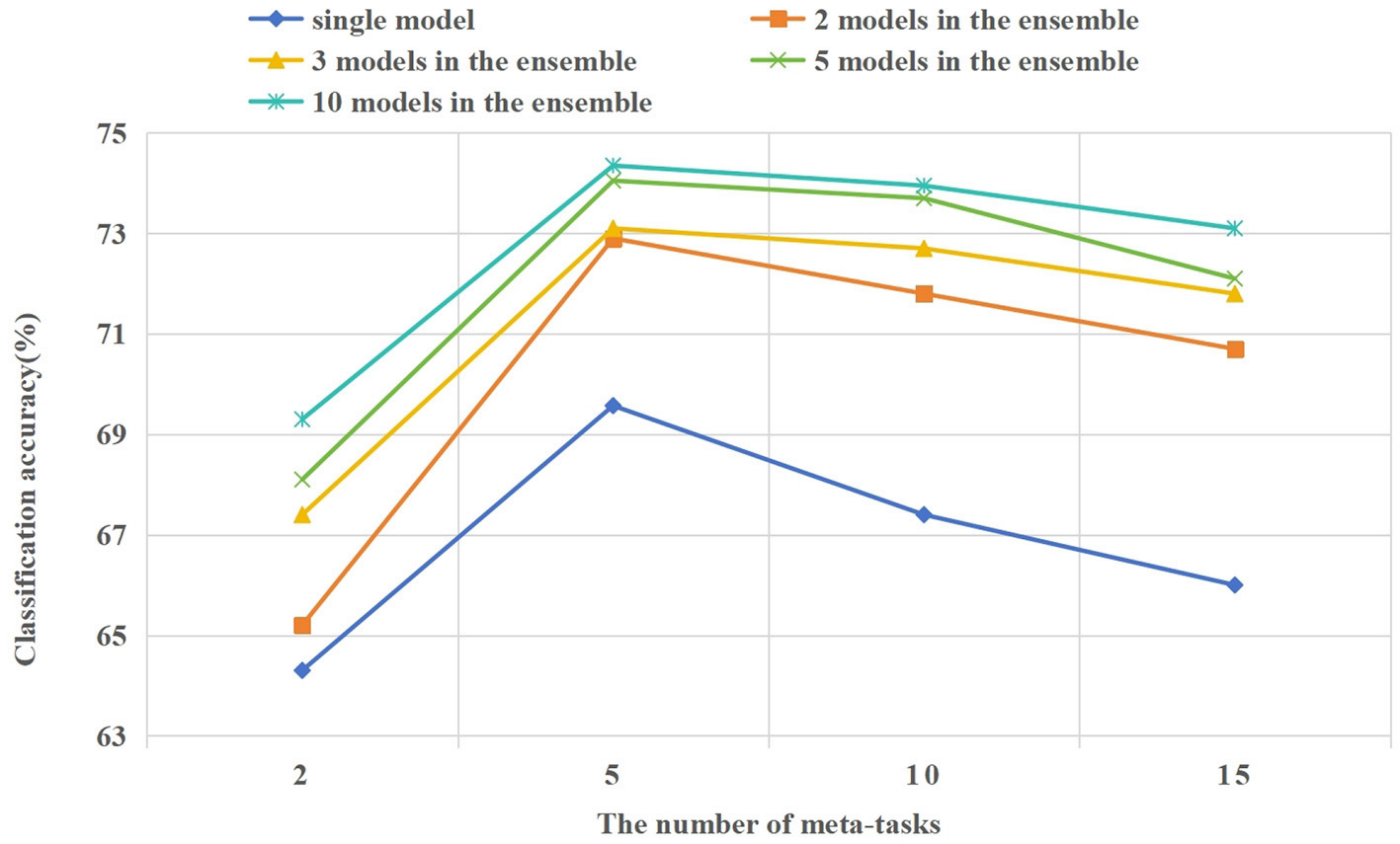
In the 5way-1shot experiment of the CIFAR-100 dataset, the effect of different numbers of tasks in the candidate meta-task set on the model classification accuracy.

In the 5way-1shot and 5way-5shot experiments of the CIFAR-100 dataset, we set the number of tasks in the candidate meta-task set to 2, 5, 10, and 15. In Figure 9, we compare the influence of different numbers of tasks in the candidate meta-task set on classification accuracy and training process. The following are the analysis of the experimental results.

As shown in Figure 9, the learning effect of the model is best when each class in the candidate meta-task sets corresponding to few-shot tasks contain 5 or 10 base class categories. When the number of tasks in the candidate meta-task sets is too few or too many, it will affect the learning effect of the model. When there are too few candidate meta-tasks, it will cause the model to overfit on few-shot tasks. When there are too many candidate meta-tasks, irrelevant meta-knowledge will be included, which will affect the learning effect of the model on few-shot tasks. Experiments show that the appropriate selection of meta-tasks from the base class is beneficial to few-shot learning.

**Figure 10:**
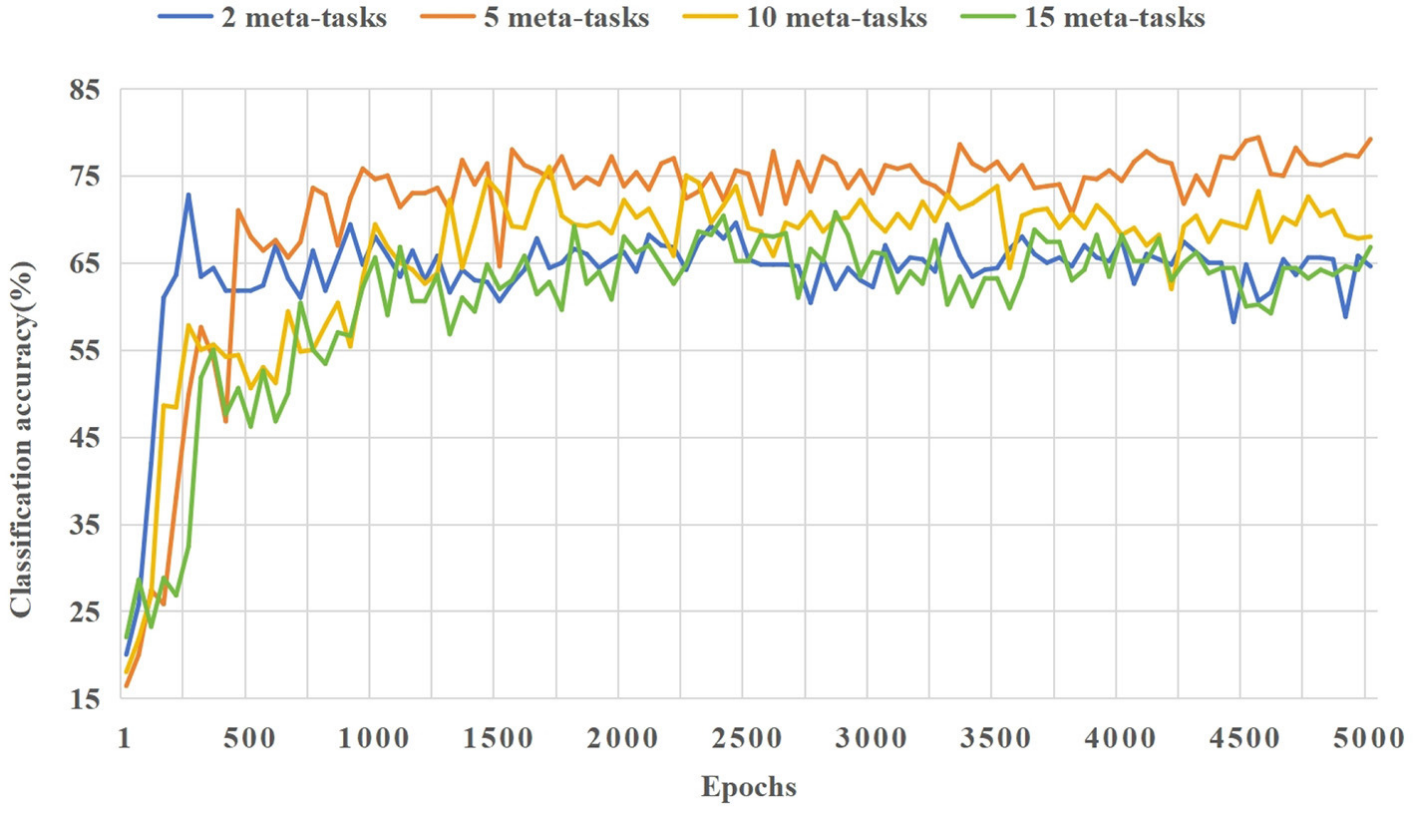
The effect of different numbers of tasks in the candidate meta-task set on the model training process in the 5way-5shot experiment of the CIFAR-100 dataset.

As shown in Figure 10, when the candidate meta-task set corresponding to each few-shot class is set to five base class categories, the training effect is the best. The model can adapt rapidly the few-shot classification tasks, and its average performance is higher than that of other cases.

### 4.5 Implementation details

We implement the FMPMIMC method based on PyTorch, which uses CNNs, Vgg16, and ResNet50 network architectures for different datasets. The network parameters are optimized by Adam optimizer. In the experiments conducted on the Omniglot dataset, setting the learning rate of the meta-learner to 10^−2^ yields the best effect, while setting the learning rate of meta-learner to 10^−3^ in experiments of the other two datasets produces the best effect. In the ensemble experiment of 20 models, the running memory exceeded 32 GB. We alleviate the problem of insufficient GPU running memory by reducing the size of the input batch of the network. All experiments are conducted on the NVIDIA Tesla V100 GPU to complete the training procedure.

## 5 Conclusion

Meta-learning methods based on optimization are widely used to improve the performance of few-shot learning. In this article, we provide a new idea for few-shot learning and study new methods for meta-task construction and multiple initial model cooperation. Considering the challenges discussed in our previous work, this article puts forward a full-phase meta-learning method based on situational meta-task construction for multiple model cooperation, which achieves few-shot learning and attempts to improve this problem. Experiments with both 5way-1shot and 5way-5shot tasks are conducted on several datasets, and the analyses prove the effectiveness of our proposed CSMT and FMPMIMC methods. Visualization experiments are more intuitive and vivid, which verifies that we provide useful meta-knowledge for few-shot tasks. The parameter setting experiments explore the influence of the iteration frequency between meta-training and meta-testing in the meta-optimization phase, the number of ensemble models, and the number of candidate meta-task set categories on training results. Our proposed methods are more suitable for providing relevant meta-knowledge to the model during the meta-training phase using novel few-shot tasks, which helps the model to learn and adapt. In future work, we will extend our methods to provide more generalized and useful meta-knowledge to the model during the meta-training period when the novel few-shot tasks are completely invisible.

## Data availability statement

The original contributions presented in the study are included in the article/supplementary material, further inquiries can be directed to the corresponding author.

## Author contributions

ZZ: Conceptualization, Methodology, Software, Writing—original draft. LZ: Data curation, Funding acquisition, Investigation, Resources, Writing—review & editing. YW: Investigation, Software, Validation, Visualization, Writing—review & editing. NW: Conceptualization, Project administration, Supervision, Writing—review & editing.
